# Detection and Prognostic Relevance of Circulating and Disseminated Tumour Cell in Dogs with Metastatic Mammary Carcinoma: A Pilot Study

**DOI:** 10.3390/cancers11020163

**Published:** 2019-02-01

**Authors:** Laura Marconato, Antonella Facchinetti, Claudia Zanardello, Elisabetta Rossi, Riccardo Vidotto, Katia Capello, Erica Melchiotti, Paola Laganga, Rita Zamarchi, Marta Vascellari

**Affiliations:** 1 Centro Oncologico Veterinario, 40037 Sasso Marconi (BO), Italy; lauramarconato@yahoo.it (L.M.); laganga@centroncologicovet.it (P.L.); 2 Veneto Institute of Oncology IOV-IRCCS, Padua, Italy; antonella.facchinetti@unipd.it (A.F.); elisabetta.rossi@unipd.it (E.R.); riccardo.vidotto@outlook.com (R.V.); 3 Department of Surgery, Oncology and Gastrenterology, University of Padova, 35128 Padova, Italy; 4 Istituto Zooprofilattico Sperimentale delle Venezie, 35020 Legnaro (PD), Italy; czanardello@izsvenezie.it (C.Z.); kcapello@izsvenezie.it (K.C.); emelchiotti@izsvenezie.it (E.M.); mvascellari@izsvenezie.it (M.V.)

**Keywords:** circulating tumour cells (CTCs), disseminated tumour cells (DTCs), animal model, metastatic breast cancer

## Abstract

In human breast cancer, both circulating tumour cells (CTCs) in peripheral blood and disseminated tumour cells (DTCs) in the bone marrow are predictive of short survival and may be used as liquid biopsy to guide therapy. Herein we investigate, for the first time, the feasibility to quantify CTCs and DTCs in canine metastatic mammary carcinoma (MMC) with the automated CellSearch platform, which identifies tumour cells by immune-magnetic enrichment and fluorescent labelling. Using this approach before start of treatment, we could detect at least 1 CTC per 7.5 mL of peripheral blood in 12 out of 27 evaluable samples (44.4%) and at least 1 DTC per 1 mL of bone marrow in 11 out of 14 evaluable samples (78.6%). Conversely, we did not find any CTCs in the healthy, negative control dogs (*n* = 5) that we analysed in parallel. Interestingly, the levels of CTCs/DTCs and the prevalence of positive dogs closely resemble results obtained by CellSearch assay in metastatic breast cancer patients at diagnosis. Moreover, in the canine cohort, the presence of CTCs was significantly associated with poor outcome. These observations identify the first actionable marker in veterinarian oncology to guide treatment of canine MMC. Furthermore, our findings have important implications for human research, since it reinforce the value of canine MMC as model useful to speed up pharmacological studies with primary endpoint of overall survival, given the reduced life-span of the canine species.

## 1. Introduction

Animal models have been extensively used in cancer research to investigate biology and genetics of human malignancies, including breast cancer, as well as efficacy and toxicity of chemical and biological agents [1]. After the recent advent of immunotherapy, much attention has been focused on animal models outbred and with preserved immune system [2]. Since dogs develop spontaneously mammary tumours, exhibiting a number of clinical and molecular similarities with human breast cancer [3], canine mammary tumour could be an excellent model to study human disease.

Mammary gland carcinomas are a very common malignancy in adult bitches.

Reported annual incidence rates range between 145 and 250 per 100,000 dogs-year at risk amongst female dogs [4,5,6,7]. In general, more than 40% of tumours in female dogs are mammary tumours (MT) [6,8,9] and approximately 30–50% of canine MT are malignant [8,10].

Regarding neutering status, it has been reported that ovariohysterectomy before the first or second oestrus cycle significantly reduces the relative risk of developing MT, while ovariohysterectomy later in life had no significant effect [8,10,11]. More recently, the authors of a systematic review concluded that due to the limited evidence available and the risk of bias in the published results the evidences that neutering reduces the risk of MT and that age at neutering has an effect, are weak [12].

Approximately 50% of canine mammary gland tumours are malignant, and 50% of these tend to infiltrate the surrounding tissues and metastasize to regional lymph nodes and lungs [13,14].

While surgery represents the main therapy with curative intent for WHO stage I–III canine mammary carcinoma, the metastatic spread to regional lymph nodes and distant sites accounts for the vast majority of cancer-related deaths [15,16]. The metastatic potential of canine mammary carcinoma is currently based on clinical stage and histopathological features of the surgical specimen, such as histotype, grade and status of the margins [17,18]. However, no reliable markers exist to predict the early development of metastases before they occur [19,20].

Circulating tumour cells (CTCs) are rare and defined as heterogeneous cells that shed from the primary tumour and circulate in the peripheral blood (PB) of human patients with cancer. Since sites of secondary disease are consistent with blood vessels’ anatomy, hematogenous dissemination of tumour cells has for a long time supposed to have been a key step of metastatic dissemination [21,22] 

Furthermore, in the last fifteen years, direct and indirect evidences contrasted the view that hematogenous dissemination of tumour cells to secondary sites is a late and minor event respect to the lymphatic dissemination, challenging the traditional model of metastasis. In fact, since bone marrow can be invaded by tumour cells only through the blood flow, it is noteworthy that at diagnosis Braun and colleagues detected micro-metastases in the bone marrow of 30.6% of human breast cancers, regardless of disease stage [23]; these tumour cells, indicated as disseminated tumour cells (DTCs), were associated with poor outcome stage [23]. 

Moreover, karyotypic abnormalities of micro-metastasis in bone marrow, both in breast cancer patients and in animal models, indicate that tumour cells disseminate already during the pre-invasive stage of disease [24], when the primary tumour is still undetectable. According to a general consensus, bone marrow can hence act as a reservoir of tumour cells, namely DTCs, which can then re-enter the circulation and spread to distant organs, finally giving overt metastasis [25].

By injecting intravenous C57BL/6J mice with radiolabelled melanoma cells cultured in vitro, Fidler and colleagues [26] extrapolated that less than 0.01% of the injected cells displayed competence to grow metastasis, since very few surviving tumour cells were needed to establish a metastasis. Several years later, the author itself provided consistent evidence in animal model that a single tumour cell may be enough to develop metastasis in vivo [27]. Indeed, although rare in human malignancies, CTCs have been associated with progression-free survival and treatment efficacy in different solid tumours, including breast cancer [28].

Moreover, CTCs have been proposed as a potential biological marker for early detection, prognosis and treatment selection and as a promising tool for monitoring disease progression, since a high number of CTCs measured at any time during treatment has been associated, in several studies, with a shorter time to progression [29,30]. Conversely, a decreasing CTC number during treatment has been associated with therapeutic success [30,31]. 

Over the past two decades, many systems for CTC/DTC detection have been developed. In 2004, CTC enumeration using the CellSearch (CS) system was shown to be significantly associated with outcome in women with metastatic breast cancer [29], leading the Food and Drug Administration to approve this technique as a method to monitor breast cancer treatment and to indicate its effectiveness. In particular, a risk threshold could be established, since human patients with ≥5 CTCs per 7.5 mL PB had a shorter progression-free survival and overall survival (OS) [29]. Notably, a pooled analysis of individual data obtained from 1944 metastatic breast cancer patients from 17 European centres confirmed that CTC enumeration at baseline is a strong independent prognostic marker that adds value to the existing clinical prognostic variables [30,31].

In veterinary medicine, only few studies have aimed, so far, to identify CTCs in the canine species [32]. Specifically in mammary carcinoma, CTCs have been detected by means of a nucleic acid-based method based on reverse transcriptase polymerase chain reaction (RT-PCR) assays [33,34]. However, with this method, no association was found between CTCs and outcome in a clinical setting.

On these bases, we designed a prospective, single-centre pilot study, whose primary objective was: to enumerate CTCs and DTCs in dogs with metastatic mammary carcinoma (MMC), using CS, before medical treatment and at the first follow-up visit. The secondary end-points were: (1) to correlate CTC and DTC enumeration with survival time (ST); (2) to establish a cut-off value of prognostic significance.

## 2. Results

### 2.1. Clinical Characteristics

Thirty-two female dogs were enrolled: 21 (65.6%) purebred and 11 (34.4%) crossbreed dogs. Among pure bred dogs, Bichon frisè (*n* = 2, 10%), Poodles (*n* = 2, 10%) and Golden retrievers (*n* = 2, 10%) were the most common. Twenty-nine (90.6%) dogs were spayed and 3 (9.4%) were intact. Median age was 11 years (range, 5–15 years) and median weight was 19.6 kg (range, 3.2–44.4 kg).

Overall, 25 (78.1%) dogs had undergone previous surgery and 7 (21.9%) dogs were considered non-surgical candidates.

At presentation, all dogs had measurable disease. Twenty-three (71.9%) had unilateral mammary cancer, whereas 9 (28.1%) had bilateral involvement.

Regarding clinical stage, 1 (3.1%) dog had stage III disease, 9 (28.1%) had stage IV disease (nodal metastasis) and 22 (68.8%) had stage V disease. The dog with stage III disease had histologically proven neoplastic emboli. Among dogs with stage V disease, 10 (45.5%) had cutaneous metastasis, 5 (22.7%) had cutaneous and pulmonary metastasis, 2 (9.1%) had cutaneous and splenic metastasis, 1 (4.5%) had cutaneous and liver metastasis, 1 (4.5%) had lung metastasis, 1 (4.5%) had cutaneous, lung and spleen metastasis, 1 (4.5%) had cutaneous, lung, spleen and liver metastasis and 1 (4.5%) had bone metastasis.

### 2.2. Histopathology

Overall, 26 out of 32 paraffin blocks were retrieved and underwent histological and immune histochemical investigations, whereas the remaining 6 samples (referred as 5 simple carcinomas and 1 anaplastic carcinoma with lymphatic invasion) were unavailable for revision. Based on the WHO classification, 3 (11.5%) samples were classified as complex carcinoma (1 grade I and 2 grade III), 14 (53.8%) as simple carcinoma (7 grade II and 7 grade III), 5 (19.2%) as anaplastic carcinoma (grade III) and4 (15.4%) as solid carcinoma (grade III).

**Figure 1:**
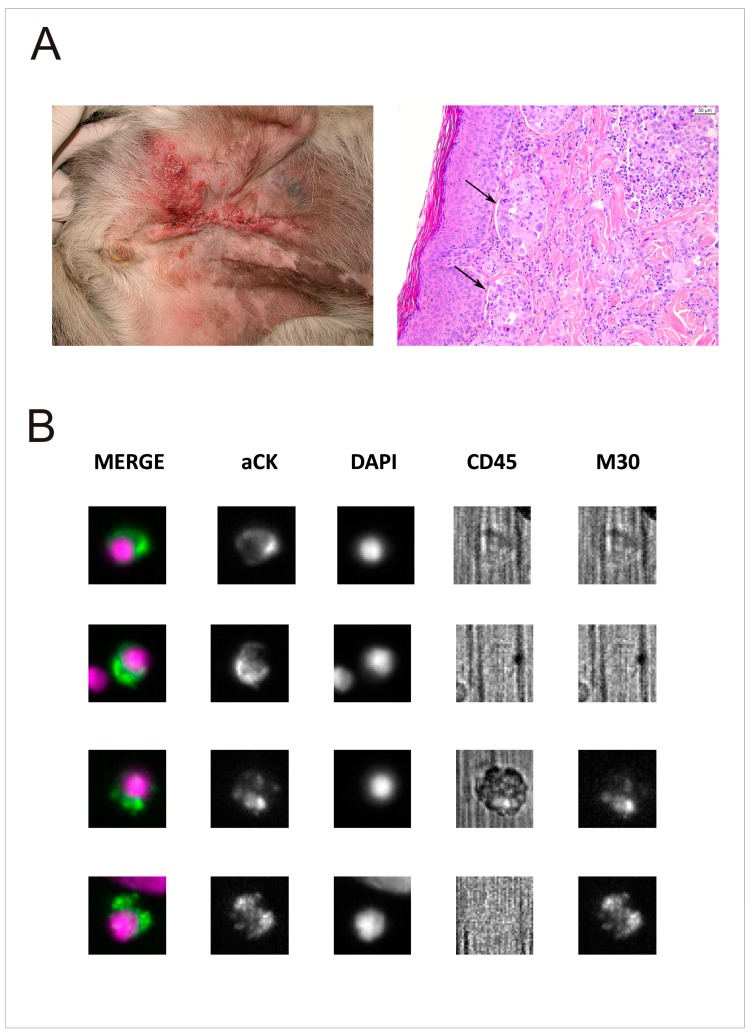
Clinical and histological characteristics and CTC/DTC enumeration of inflammatory carcinoma of dog. (**A**) A representative case of MMC (DOG-16), out of 10 is shown. On the left, mixed breed dog, 11 years old, spayed female, affected by inflammatory mammary carcinoma. On the right, in skin section sheets of anaplastic carcinoma, epithelial cells infiltrating the dermis and invading lymphatic vessels (arrows) are evident. Haematoxylin and eosin stain, 20× magnification. (**B**) Analysis of 4 out of 16 rare cells detected in baseline BM sample of DOG-16, using an Analyzer II device (Menarini). Horizontally, the photos show the same cell stained for the combination (MERGE) of CK (green) and DAPI (violet); CK PE only; DAPI only; CD45 APC only; and M30 FITC only. Based on M30 staining profile (sufficient signal relative to background) we classified the last 2 cells as M30-positive (apoptotic) DTCs. We did not find any CTCs in the PB collected at the same time of BM sample.

Eleven out of 26 tumours (42.3%) were further classified into the clinical-pathologic entity of inflammatory mammary carcinoma, based on the presence of neoplastic emboli within the lymphatic vessels of the dermis (Figure 1A).

By immunohistochemistry, 6 (23.1%) samples were positive for ERα, 6 (23.1%) were positive for HER-2, 1 (3.8%) was positive for PR and 1 (3.8%) sample showed co-expression of ER/PR, whereas 12 out of 26 (46.2%) samples were negative for all three antibodies tested.

### 2.3. Treatment and Response

Thirty out of 32 dogs (93.8%) received medical treatment, consisting of a tyrosine kinase inhibitor (*n* = 18) or systemic cytotoxic chemotherapy (*n* = 12). Two (6.2%) dogs died shortly after the pre-treatment evaluation and received no treatment.

Among the 30 dogs that were evaluable for treatment response, 5 (16.7%) achieved complete response (CR), defined as disappearance of all lesions for >4 weeks; 6 (20%) achieved partial response (PR), defined as a decrease of at least 30% in the diameter of a lesion for >4 weeks; 2 (6.7%) were stable (<30% reduction or 20% increase in the diameter of a lesion); and 17 (56.7%) progressed, (appearance of a new metastatic lesion or at least a 20% increase of the diameter of a lesion).

At the end of the study, 25 dogs had died (21 due to their cancer and 4 for unrelated causes), 5 were still alive and 2 dogs were lost at follow-up. The median follow-up time was 167 days (25th percentile: 97 days, 75th percentile: 315 days) while the median ST was 189 days (25th percentile: 104 days, 75th percentile: 454 days). The 4 dogs that had died for unrelated causes were censored.

### 2.4. CTC and DTC Prevalence in MMC

Overall, 32 and 19 dogs had PB and BM drawn at baseline (T1) for CTC and DTC enumeration, respectively. At the first follow-up (T2), 15 and 6 dogs underwent CTC and DTC enumeration, respectively. The number of evaluable dogs at the following time point decreased because of death, occurring in the meantime.

Considering the feasibility of CS assay in the veterinary setting, at T1 informative results were obtained in 27 out of 32 (84.4%) PB samples and in 13 out of 19 (68.4%) BM samples, respectively. At T2, informative results were obtained in 15 out of 15 (100%) PB samples and in 3 out of 6 (50%) BM samples. The main reasons for unsuccessful tests were partially clotted samples or slides not fully satisfying internal quality controls (at least two DAPI+ events for doing autofocus, in at least 6 out of 9 selected microscopic fields), so that the automated scanner of the slide failed.

At T1, different levels of tumour cells in PB and BM were documented. Indeed, 12 out of 27 (44.4%) dogs had at least 1 CTC/7.5 mL PB and 11 out of 14 (78.6%) dogs had at least 1 DTC/1 mL BM, respectively (Figure 1B). Conversely, we did not find any CTCs in PB of healthy, negative control dogs (*n* = 5).

The expression of M30, a marker of early apoptosis in epithelial cells, was high both in PB and in BM samples, with a median value of 75% and 90% in CTCs and DTCs, respectively and high inter-samples variability (range 0 to 100%, in both districts).

### 2.5. Prognostic Significance of CTC/DTC Enumeration

Table 1 summarizes the distribution of clinical-pathological characteristics of the cohort, according to the presence of CTCs. For all but one variables (the expression of progesterone receptor), we had more than 1 case, hence we could perform statistics, by appropriate tests for a pilot study (Chi-square test with Yates correction or Fisher’s exact test).

Overall, CTCs were not associated with clinical-pathological characteristics (Table 1), including the presence of single versus multiple sites of metastasis. Notably, prior surgery and neutering status did not affect CTC prevalence at baseline.

Inflammatory carcinoma is the only remarkable exception, since it was significantly associated with the occurrence of CTCs (Table 1, *p* = 0.035, Fisher’s exact test).

At T2, only 4 out of 15 (26.7%) evaluable PB samples were CTC-positive, meanwhile 2 out of 3 (66.7%) evaluable BM samples resulted DTC-positive. By comparing T1 and T2 results, 6 out of 8 (75%) samples that were CTC-negative at T1 remained negative at T2, while 5 out of 6 (83%) positive samples at T1 turned out to be negative at T2. The dogs of this last group were too few to do further speculations, however, we recorded that 4 out of these 5 dogs died because of mammary cancer after 50, 72, 189 and 294 days, respectively, whilst the fifth dog was alive at data analysis closure after 523 days.

Concerning changes in DTC levels, in paired T1–T2 samples we recorded not evaluable, 8 and 41 cells at T1 versus 2, 0 and 8 DTCs at T2, in cases #21, #22 and #24 respectively. 

Moreover, in both PB and BM, the levels of tumour cells decreased at T2, with a median number of 1 CTC and 2 DTCs, respectively.

**Figure 2:**
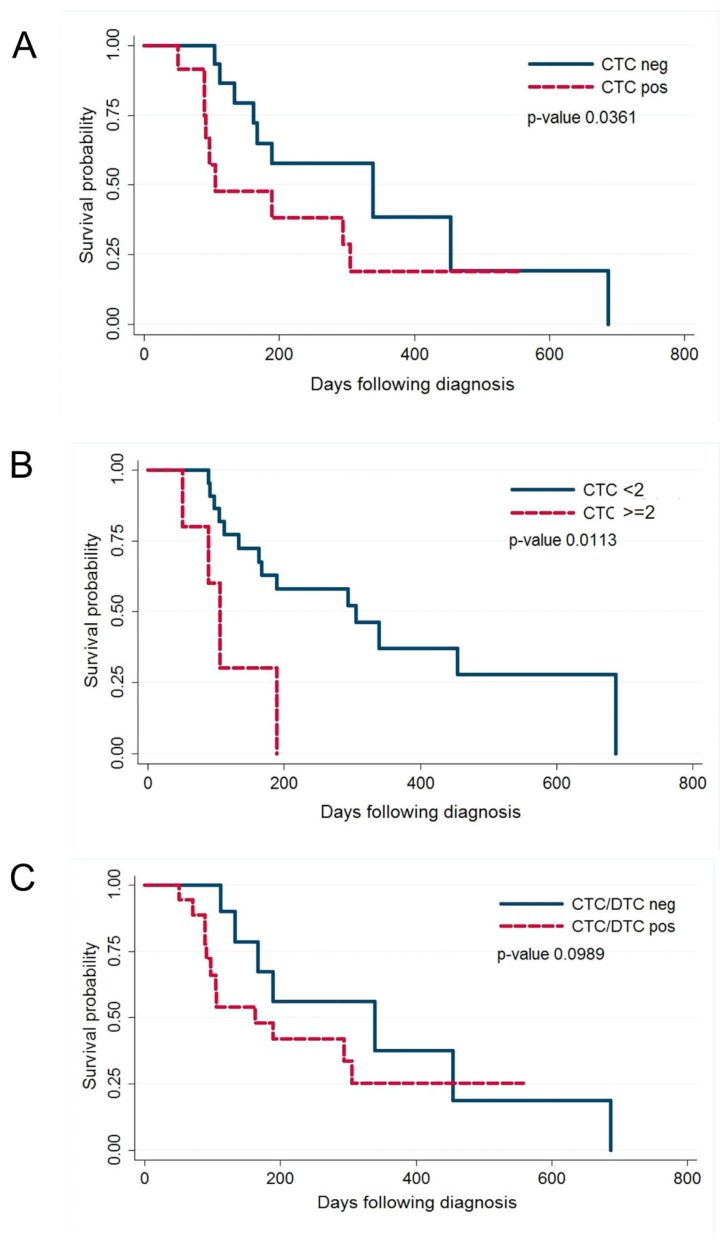
Kaplan–Meier (KM) estimates of probabilities of Survival Time (ST) in dogs with MMC. (**A**) KM for dogs with <1 CTCs per 7.5 mL of PB and those in the group with ≥1 CTCs per 7.5 mL of PB before initiation of therapy; (**B**) KM for dogs with <2 CTCs per 7.5 mL of PB and those in the group with ≥2 CTCs per 7.5 mL of PB before initiation of therapy; (**C**) KM for dogs with <1 CTCs and DTCs and those in the group with at least ≥1 CTCs or DTCs before initiation of therapy.

By stratifying dogs according to CTCs measured at T1 (*n* = 27), a significant association with ST was documented; indeed, the median ST was 339 days in CTC-negative dogs (*n* = 15) versus 105 days in dogs with at least 1 CTC (*n* = 12) (*p*-value *=* 0.0361, Wilcoxon test; Figure 2A). Considering a cut-off of CTCs ≥ 2, a stronger association with ST was observed (305 vs.105 days for CTC numbers < 2 vs. ≥ 2, respectively) (*p*-value = 0.0113, Wilcoxon test; Figure 2B). Conversely, the DTC count at T1 did not show any significant association with ST (*p*-value = 0.8299, Wilcoxon test), even though dogs with at least 1 DTC (*n* = 11) showed shorter median ST (189 days) than DTC-negative dogs (*n* = 3; 454 days).

Finally, the presence of at least 1 cell, regardless of the site (PB or BM) showed a weak association with ST (*p*-value = 0.0989, Wilcoxon test; Figure 2C).

## 3. Discussion

In dogs, MMC represents a therapeutic challenge, mainly due to the very limited treatment options. Once distant metastases have occurred, the disease remains largely incurable, with a median ST that rarely exceeds a couple of months. While this holds true for the majority of dogs, the risk of outcome is not identical among given individuals. Acknowledging the paucity of effective treatment options for dogs with MMC, the identification of prognostic markers is clearly crucial to identify those dogs that may harbour a better prognosis and benefit from treatment, thereby guiding the clinical decision-making process.

Herein, we document for the first time the feasibility to quantify CTCs and DTCs in canine MMC with the automated CS platform. Sampling of PB and BM was uneventful and less invasive than multiple biopsies of metastatic sites and the use of an automated platform permitted the serial monitoring of the enrolled dogs.

Secondly, the levels of CTCs/DTCs and the prevalence of positive dogs closely resemble the results obtained by CS in human metastatic breast cancer (MBC) at diagnosis [29], further extending the similarities between humans and dogs regarding mammary tumour biology [3]. Indeed, our results demonstrate that MMC is a systemic disease that involves BM in the great majority of cases, whilst the presence of CTCs is associated with a worse outcome. Therefore, CTC enumeration in canine MMC may yield prognostic information once the tumour is first diagnosed, since the provisional cut-off of at least 2 CTCs was significantly associated to a shorter median ST (105 days). By contrast, dogs with less than 2 CTCs had a median ST of 305 days, supporting the role of CTC assay to identify those subsets of dogs that would benefit from medical treatment.

In our study, CTC enumeration resulted to be an independent prognostic factor, not related with other pathological and clinical data, such as tumour type, histological grading and clinical staging. Although this finding might be considered with caution, because of the small sample size of our pilot study, and it warrants further investigations, it is noteworthy that it is similar to what it has been observed in more than two thousand human MBC [29,31]. 

Interestingly, one third of MMCs included in this pilot study were inflammatory carcinomas and 70% of them were CTC-positive, a condition that closely resembles what has been observed in humans, in whom inflammatory breast cancer is a rare disease, burdened by early metastatic dissemination, high CTC levels and poor prognosis [35]. 

Moreover, when we analysed the expression of M30, a marker of epithelial apoptosis, we found a high level of apoptosis of tumour cells both in PB and in BM, similarly to the findings previously reported in newly diagnosed, treatment-naive metastatic solid tumours [36]. The expression of apoptosis markers in tumour cells might seem to be counterintuitive, especially when analysing metastatic disease; however, a higher histological grade and an increased proliferation are often associated with tumour necrosis and apoptosis, which we may regard as adverse prognostic features [37]. In our canine cohort, this finding is consistent with the advanced stage of the MMCs included.

In human MBC, CTC enumeration over time is important in treatment monitoring [30]. We can also confirm in our canine cohort that the CTC/DTC assay is dynamic enough to document decreasing levels of CTCs and DTCs at the first follow-up visit. Herein, we analysed all cases cumulatively, regardless of the type of treatment, since the primary objective of the study was to test the feasibility of the automated test and the sample size was accordingly small. The determination of the better therapeutic choice was beyond the purpose of this pilot study, an objective that warrants evaluation in larger cohorts, in ad hoc planned protocols [38]. Nevertheless, based on our findings, CTC assay looks like a promising marker of treatment efficacy also in dog. 

The main limit of our study is the low number of samples that prevented complete statistical analysis. In addition, the number of controls, close to 15% of the full cohort entered in the study, is low because of ethical reasons. However, the findings herein reported have to be considered a first milestone for further investigations. 

At this regard, the heterogeneity of the population enrolled in this pilot study (as for breed, treatments and neutered status) mimics that of human population and it might be a plus, in view of potential application in translational research on human cancer.

In addition, since sampling the iliac crest allows, as opposed to other sites (including the humerus or ribs), a better and safer animal restraint, we could examine PB and BM in parallel, even if in few cases only. This is of utmost importance for translational studies, since bone marrow screening for occult metastatic tumour cells has not been included, in human cancer, as standard clinical routine in the majority of the European Member States [39], because of the procedure’s invasiveness [40]. 

In conclusion, our study documents that CTCs and DTCs are an actionable prognostic biomarker of canine MMC, as the minimal invasiveness of sampling procedures and the use of an automated platform enable high robustness and reproducibility of findings. Taken together, these two aspects will lead to the design of prospective studies to evaluate treatment efficacy in veterinary oncology.

## 4. Methods

### 4.1. Animals and Samples

The study protocol was approved by Committee of the “Istituto Zooprofilattico Sperimentale delle Venezie (IZSVe)” (ethic code: # 18913-P) responsible for animal protection and welfare and a mandatory written consent from all dog’s owners was obtained. All the experiments were performed in accordance with the relevant guidelines and regulations, namely the Italian D.L. no. 26, 4 March 2014 that implements the European Directive 2010/63/UE regarding the protection of animals used for experimental and other scientific purposes and the Good Clinical Practice.

Overall, between December 2014 and January 2018, 32 client-owned, treatment-naïve dogs with a clinical and histopathological diagnosis of measurable MMC were eligible for recruitment. 

We further analysed 5 PB samples from healthy dogs (from December 2014 and January 2019), which served as negative controls for CTC detection. Dogs used as negative controls were healthy female dogs, belonging to the blood donor register of the Istituto Zooprofilattico Sperimentale delle Venezie, that underwent periodically medical and laboratory screening, to exclude pathological status. The complete signalament of controls was: 1 Golden retriever, neutered female, 8 years old; 1 Newfoundland, intact female, 3 years old; 1 Labrador neutered female, 5 years old; 1 mixed breed, neutered female, 7 years old; 1 American Staffordshire terrier, 2 years, entire female. We did not sample the BM for ethical reasons. 

Study inclusion criteria were as follows:

1. At least one unidimensional lesion measurable by ultrasound or computed tomography (CT), according to the canine Response Evaluation Criteria In Solid Tumours version 1.0 (cRECIST v1.0) [41];
2. Clinically detectable metastatic disease or the presence of lymphatic emboli, confirmed by histopathology;
3. Prior surgery without systemic chemotherapy permitted.

A further optional inclusion criterion was:

4. Availability of tumour tissue for histopathological evaluation and immune-histochemical staining.

Exclusion criteria were previous systemic chemotherapy or molecular target therapy.

At enrolment, all dogs underwent a complete routine staging work-up, consisting of tumour biopsy, CT or abdominal ultrasound and thoracic radiographs. 

Additionally, a clinician was asked to collect into CellSave tubes (Menarini-Silicon Biosystems, Castel Maggiore, BO, Italy) both PB (7.5 mL) and BM (2 mL) of dogs from the iliac crest. In dogs, the iliac crest is commonly used for BM aspiration due to the greater feasibility compared to other sites. The dogs were not anesthetized for the procedure and sampling the iliac crest allowed as opposed to other sites (including the humerus or ribs) for a better and safer animal restraint.

Subsequently, we maintained samples at room temperature during the transport to the CTC-lab of IOV-IRCCS, where we performed baseline CTC/DTC enumeration within 96 h from the blood draw, according to manufacturer′s instructions.

The type of treatment was at the investigator’s personal discretion and included traditional cytotoxic chemotherapy or target therapy and surgery when indicated. 

Reassessment of disease status was conducted at the first follow-up (T2; 6–8 weeks, depending on treatment type and schedule) with clinical examination and imaging using cRECIST criteria [41], without knowledge of baseline CTC/DTC results. 

Briefly, disappearance of all lesions for >4 weeks was defined as complete response (CR); a decrease of at least 30% in the diameter of a lesion for >4 weeks was defined as partial response (PR); the appearance of new MCTs or at least a 20% increase of the diameter of a lesion, was defined as progressive disease (PD). Less than 30% reduction or 20% increase in the diameter of a lesion, was defined as stable disease (SD). 

### 4.2. Histopathological Analysis

Surgical or bioptic paraffin-embedded samples of the cases included were retrieved by different histopathological services and further sections were stained with haematoxylin and eosin (HE) for histological evaluation. Tumours were classified according to the WHO international classification of mammary tumours of domestic animals [17,42] and grading was applied according with Peña and colleagues [18] with the agreement of two pathologists (CZ, MV).

In addition, 3-μm sections from each sample were stained by the BenchMark ULTRA automated immunostainer (Ventana Medical Systems, Tucson, AZ, USA) using 3 different antibodies (already validated in dogs by Jaillardon and coll. [43]): 

(a) The anti-progesterone receptor monoclonal rabbit pre-diluted antibody (clone 1E2, Roche Diagnostics) for 24 min at 37 °C;

(b) The Pathway anti/HER-2/neu (HER-2) monoclonal rabbit pre-diluted antibody (clone 4B5, Roche Diagnostics) for 32 min at 36 °C;

(c) The anti-oestrogen-α receptor monoclonal mouse antibody (Clone C311, Santa Cruz Biotechnology, Dallas, TX, USA) applied at 1:50 dilution for 44 min at room temperature. 

For all the three antibodies, dewaxing was performed by the instrument at 72 °C for 8 min. Antigen retrieval was performed using a commercial pre-diluted solution (pH 8.4) (ULTRA cell conditioning solution (CC1), Ventana Medical System, Tucson, AZ, USA), at 95 °C for 64 min for PR and for 52 min for HER-2. No antigen retrieval was applied for ERα antibody. 

An indirect biotin-free system (UltraView universal DAB detection kit (052 697 806 001 code), Ventana Medical Systems, Tucson, AZ, USA) was used as detection system. Haematoxylin was used as contrast stain. 

The immunohistochemical protocols for all antibodies were developed at the Laboratory of Histopathology of the Istituto Zooprofilattico Sperimentale delle Venezie using positive controls in each run, consisting of a canine mammary gland for PR and ERα antibodies and a control slide pathway for HER2 provided by the Company. Each run also included negative controls obtained by omitting the primary antibody during the labelling steps.

### 4.3. CTCs/DTCs Enumeration

The presence of CTCs and DTCs in canine PB or BM, respectively, was assessed by CellSearch^™^ System (Menarini-Silicon Biosystems, Castel Maggiore (BO), Italy), according to the manufacturer′s instructions, with minimal modification [28,29]. 

Since it has been previously reported the cross-species reactivity man/dog of monoclonal antibodies (mAbs), in particular for highly conserved proteins as cytokeratins and EpCAM [44,45], we used the standard CTC kit (Menarini), developed by the manufacturers for human samples and based on immune-magnetic enrichment and fluorescent labelling. The CellSearch platform is an automated, closed system, whose reagents are provided in a ready-to-use package that includes the following mouse mAbs: ferrofluid-conjugated anti-EpCAM, PE-conjugated anti-cytokeratins (8, 18 and 19) and APC-conjugated anti-CD45; DAPI is also included as nucleic acid dye.

Secondly, we examined a median volume of 7.5 mL PB and 1 mL BM for CTC and DTC samples, respectively; volume variations depended on the dogs’ weight, as previously reported for mouse model [46]. Finally, we adjusted the red blood cell level by diluting canine blood draws with run’s buffer, according to the maximum permitted by the CellSearch platform.

The classification of cells, instead, fully satisfied the standard CellSearch criteria. Briefly, we classified an event as a CTC, when its morphological features were consistent with those of a tumour cell and the phenotype EpCAM+, CK+, DAPI+ and CD45- was exhibited.

We also quantified apoptotic CTCs by integrating the standard assay with an anti-M30 mAb (ALX-804-590, Alexis Biochemicals, San Diego, CA, USA); M30 is a neoepitope disclosed by caspase cleavage of cytokeratin 18 (CK18) in early apoptosis of epithelial cells [36]. Quantitative results were expressed as the total number of cells and of M30-positive cells per 7.5 mL PB or 1 mL BM, for CTCs and DTCs, respectively.

### 4.4. Statistical Analysis

The association with CTC level was assessed using Chi-square test with Yates’ correction or Fisher′ exact test for clinical-pathological characteristics and Student *t*-test for age distribution.

The Kaplan-Meier method was used to draw the cumulative survival curves, applied to time between the date of diagnosis and the date of death for mammary tumour; the dogs alive at the end of follow-up period or those dead for other causes were censored using the time between the date of diagnosis and their most recent follow-up evaluations.

The proportional-hazards assumption was evaluated by means of a log-log plot. Then, the Wilcoxon test was adopted [47] to compare survival curves between MMC groups, defined as: (a) CTC ≥ 1 and CTC < 1 cell; (b) CTC ≥ 2 and CTC < 2 cells; (c) DTC ≥ 1 and DTC < 1 cell; and (d) dogs with CTC or DTC ≥ 1 versus dogs with a cell number < 1 cells for both CTC and DTC.

All statistical analyses were carried out using STATA version 12.1.

## 5. Conclusions

Finally and importantly, our findings have a translational interest, since canine invasive mammary tumours share many features with human breast cancer and develop spontaneously in dogs with an intact immune system, without genetic or chemical manipulation [3]. The value of this opportunity has been increasingly recognized in different fields of cancer research, such as tumour biology and cancer progression, identification of cancer-associated genes and the development of novel cancer therapeutics. To similar purposes, CTC derived mouse models (CDXs) have been proposed by several authors in malignancies of lung [48,49], liver [50], breast [46,51] and prostate [46], among the others. However, as the patient-derived mouse models (PDXs) [52], CDXs have low success rate, require long time to be established and are unsuitable to investigate tumour/microenvironment and tumour/immune system interplay. 

Herein, we show that the model of canine MMC, monitored by dog CTCs, addresses these challenges, in particular for clinical trials purposes, since the shorter lifespan of dogs with respect to humans represents an excellent resource for testing new therapeutics in a pre-clinical setting. Our study reinforces the validity of dogs as spontaneous models for cancer, highlighting further similarities in tumour spread and progression between dogs and humans. 

## Figures and Tables

**Table 1:** Clinical-pathological characteristics of dogs’ cohort.

Variable	N	CTC-Neg	CTC-Pos	*p*-Value
Breed				
Purebred	18	11 (61.11%)	7 (38.89%)	0.448
Crossbreed	9	4 (44.44%)	5 (55.56%)	
Age (years)	27	10.53 (SD * 1.99)	11.16 (SD * 2.72)	0.492
Weight (kg)	27	20.83 (SD * 13.87)	17.23 (SD * 11.33)	0.475
Previous surgery				
Non-surgical candidates	6	3 (50.00%)	3 (50.00%)	1.000
Prior surgery	21	12 (57.14%)	9 (42.86%)	
Neutering status				
Intact	3	2 (66.67%)	1 (33.33%)	1.000
Neutered	24	13 (54.17%)	11 (45.83%)	
Grading				
1–2	8	3 (37.50%)	5 (62.50%)	0.204
3	16	11 (68.75%)	5 (31.25%)	
Inflammatory carcinoma				
No	14	11 (78.57%)	3 (21.43%)	0.035
Yes	10	3 (30.00%)	7 (70.00%)	
Staging				
III + IV	10	5 (50.00%)	5 (50.00%)	0.706
V	17	10 (58.82%)	7 (41.18%)	
Metastatic sites				
Single	12	6 (50.00%)	6 (50.00%)	1.000
Multiple	14	8 (57.14%)	6 (42.86%)	
Oestrogen receptor				
Negative	18	10 (55.56%)	8 (44.44%)	1.000
Positive	6	4 (66.67%)	2 (33.33%)	
HER-2				
Negative	18	11 (61.11%)	7 (38.89%)	0.665
Positive	6	3 (50.00%)	3 (50.00%)	
Progesterone receptor				
Negative	23	13 (56.52%)	10 (43.48%)	na **
Positive	1	1 (100.00%)	0 (0.00%)	

* SD: Standard Deviation; ** na: not applicable.
